# Revealing missing charges with generalised quantum fluctuation relations

**DOI:** 10.1038/s41467-018-04407-1

**Published:** 2018-05-22

**Authors:** J. Mur-Petit, A. Relaño, R. A. Molina, D. Jaksch

**Affiliations:** 1 Clarendon Laboratory, University of Oxford, Oxford, OX1 3PU UK; 2 Departamento de Física Aplicada I and GISC, Universidad Complutense de Madrid, Av. Complutense s/n, 28040 Madrid, Spain; 3 Instituto de Estructura de la Materia, IEM-CSIC, Serrano 123, 28006 Madrid, Spain; 4 Centre for Quantum Technologies, National University of Singapore, Singapore, 117543 Singapore

## Abstract

The non-equilibrium dynamics of quantum many-body systems is one of the most fascinating problems in physics. Open questions range from how they relax to equilibrium to how to extract useful work from them. A critical point lies in assessing whether a system has conserved quantities (or ‘charges’), as these can drastically influence its dynamics. Here we propose a general protocol to reveal the existence of charges based on a set of exact relations between out-of-equilibrium fluctuations and equilibrium properties of a quantum system. We apply these generalised quantum fluctuation relations to a driven quantum simulator, demonstrating their relevance to obtain unbiased temperature estimates from non-equilibrium measurements. Our findings will help guide research on the interplay of quantum and thermal fluctuations in quantum simulation, in studying the transition from integrability to chaos and in the design of new quantum devices.

## Abstract (web summary)

Conservation laws are a key ingredient in the non-equilibrium dynamics of quantum many-body systems. Here, the authors develop generalised quantum fluctuation relations in order to identify the presence of conserved quantities relevant for a generalised Gibbs ensemble.

## Introduction

Einstein famously vouched for the enduring success of thermodynamics ‘within the framework of the applicability of its basic concepts’ stemming from the simplicity of its premises and breadth of its scope^1^. From its birth as a practical science in the cradle of the industrial revolution^2^ to modelling thermal fluctuations in biological processes through fluctuation relations (FRs)^3,4^, thermodynamics constitutes one of the most successful theories to understand nature. The increasing degree of control on meso- and nano-scopic systems has driven interest into the field of quantum thermodynamics to describe phenomena where both quantum effects and finite-size fluctuations are apparent^5^. Important findings so far range from generalised Carnot bounds on the efficiency of quantum heat engines^6–8^ to quantum versions of the classical FRs—i.e., quantum FRs (QFRs)—for processes starting in a canonical equilibrium state^9^. According to the principles of quantum statistical mechanics, such a state is characterised by a single parameter, the inverse temperature *β*, which also plays a special role in the QFRs (see ref. ^10^ and references therein).

The dynamics of an important number of quantum systems, however, eludes this approach. Integrable quantum systems, for instance, feature a large number of conserved quantities, or ‘charges’, which effectively constrain the phase space that the system can explore in its dynamic evolution^11^. Notable models with charges include the one-dimensional Hubbard model^12^, the Dicke model^13,14^ and the super-symmetric *t*-*J* model^15^, to name a few. The existence of charges leads sometimes to striking experimental observations such as the practically dissipationless dynamics of the quantum Newton’s cradle^16^, which stems from the (infinitely many) charges of the Lieb–Liniger model. On other occasions, however, their existence is far from obvious. For instance, it is only recently that a whole set of quasi-local charges in XXZ model were discovered^17–19^ as result of a discrepancy observed between numerical simulations of the model and estimates based on the Mazur bound^20^.

It is a critical task of quantum many-body physics to develop methods to confidently ascertain whether a quantum system features such charges, especially when these may be difficult to measure directly. Unfortunately, the Mazur bound is only sensitive to charges with a non-zero overlap with the current operator, which implies it cannot serve as a general witness to unveil all charges in a generic quantum many-body system. It is thus necessary to develop more general systematic methods to explore the existence of unknown conserved charges in quantum many-body systems.

Here we introduce an approach to this problem based on a statistical analysis of arbitrary non-equilibrium measurements of the system of interest. In short, we demonstrate that non-equilibrium measurements on a quantum many-body system are more sensitive to the existence of charges than equilibrium ones, and describe a protocol that exploits this sensitivity to reveal the existence of charges that restrict the dynamics in any degree of freedom of the system. To this end, we first provide a theory that completely characterises the non-equilibrium fluctuations of quantum systems with conserved quantities. Specifically, we present generalised versions of the Tasaki–Crooks relation (TCR)^21^ and the quantum Jarzynski equality (QJE)^22–24^ suitable to describe fluctuations in systems with an arbitrary, and possibly variable, number of charges. Then, we show how these results open the door to determining from experimental measurements the existence of hitherto unknown charges. We also discuss how this can improve the accuracy, e.g., of temperature measurements. Finally, we illustrate our results with simulations of the Dicke model^13, 14^, a well-known many-body model that features a single charge and which can be realised in current experimental platforms.

## Results

### Theoretical framework

**Fig. 1:**
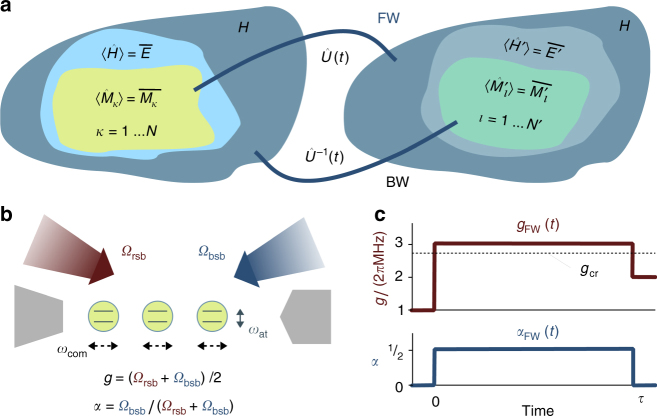
Sketch of the system and protocol. (**a**) The dynamics of a generic quantum system with average energy $\left\langle {\hat H} \right\rangle = \overline E$ occurs within a restricted subspace (light blue area) of its full Hilbert space, ${\cal H}$ (dark blue). If additional conserved quantities exist, the dynamics is further restricted to a smaller subspace (yellow). An equilibrium state of such a system with charges is described by a generalised Gibbs ensemble density matrix, Eq. (1). Here, we consider two unitary processes $\hat U(t),\hat U^{ - 1}(t)$ that drive the system out of two such equilibrium states corresponding to Hamiltonians $\hat H$ and $\hat H\prime$, respectively. **b** Trapped ion setup: *N* ions (circles) equally coupled to a phonon mode (black arrows) are illuminated by fields addressing the red and blue sidebands (wide red and blue arrows) with Rabi frequency *Ω*_rsb_[*Ω*_bsb_]. **c** Time dependence of the Dicke model parameters in the forward (FW) protocol, with a variable wait time *τ* between two quenches

The quantum statistical description of systems with charges can be reliably built on Jaynes’ information theory formulation of statistical mechanics^25^. In this approach, a new statistical ensemble, the generalised Gibbs ensemble (GGE), has been proposed^26^ to incorporate the constraints on the known values of the charges to the equilibrium state via the maximum entropy principle (see also ref. ^27^). In the GGE, the equilibrium state of a system with Hamiltonian $\hat H$ is given by a density matrix of the form

1 $$\hat \rho _{{\mathrm{GGE}}} = \frac{1}{{\cal Z}}{\mathrm{exp}}\left( { - \beta \hat H - \mathop {\sum}\limits_{k = 1}^{\cal N} {\kern 1pt} \beta _k\hat M_k} \right),$$

where ${\cal Z} \equiv {\cal Z}\left( {\vec \beta ,\hat H,\left\{ {\hat M_k} \right\}} \right)$ = ${\mathrm{Tr}}\left[ {{\mathrm{exp}}\left( { - \beta \hat H - \mathop {\sum}\nolimits_k \beta _k\hat M_k} \right)} \right]$ is the partition function, and the operators associated with the charges, $\hat M_k$, satisfy $\left[ {\hat M_k,\hat H} \right] = 0$, for *k* = 1, …, ${\cal N}$, with ${\cal N}$ the number of charges of the system, see Fig. 1a. (Below, we will assume the charge operators commute with each other, which enables measuring them simultaneously.) The generalised inverse temperatures, $\vec \beta = \left( {\beta ,\left\{ {\beta _k} \right\}_{k = 1}^{\cal N}} \right)$, are fixed by requiring that averages over $\hat \rho _{{\mathrm{GGE}}}$ reproduce the known average values of the energy, $\left\langle {\hat H} \right\rangle \equiv {\mathrm{Tr}}\left[ {\hat H\hat \rho _{{\mathrm{GGE}}}} \right] = \overline E$, and charges, $\left\langle {\hat M_k} \right\rangle = \overline M _k$. A crucial open question springing from Eq. (1) is the identification of all charges $\hat M_k$ relevant to the dynamics of the system. The usual approach consists in the study of equilibrium expectation values of certain observables. However, this approach suffers from a number of caveats and difficulties, like the very need of measuring a large number of observables, or the existence of particular observables that may not thermalise (see Supplementary Note 1 for more details). We show below that measurements in non-equilibrium processes are highly sensitive to the existence of charges, and how one can use them to reveal the presence of conserved quantities in the equilibrium state.

### Generalised QFRs

We study the energy fluctuations of a system with charges by considering two processes (forward (FW) and backward (BW)) that take the system away from initial equilibrium states (Fig. 1a). Each process is a four-step protocol similar to the two-projective-measurement (TPM) protocol utilised to derive the standard QFRs^28^.

In the FW process, the system is (i) prepared in the equilibrium state corresponding to Hamiltonian $\hat H$. If this Hamiltonian features a number ${\cal N}$ of charges, this state can be written in the form of Eq. (1) with ${\cal N}$ + 1 parameters $\vec \beta$ = {*β*, *β*_1_, …, $\beta _{\cal N}$}. We build a basis of the Hilbert space with eigenvectors $\left| {\vec i} \right\rangle$ = $\left| {i_0,i_1, \ldots ,i_{\cal N},\eta } \right\rangle$, where the quantum number *i*_0_ identifies the energy eigenvalue, $\hat H\left| {\vec i} \right\rangle = E_{i_0}\left| {\vec i} \right\rangle$, and *i*_*k*_ similarly labels the eigenvalues of $\hat M_k$ through $\hat M_k\left| {\vec i} \right\rangle = M_{k,i_k}\left| {\vec i} \right\rangle$ (*k* = 1, …, ${\cal N}$); *η* contains the additional quantum numbers required to fully determine a basis state. After this preparation stage, (ii) at time *t* = 0 one performs simultaneous projective measurements of $\hat H$ and $\hat M_k$ on the system, obtaining definite values for its energy, ${\cal E}_{{\mathrm{ini}}}$ ∈ {*E*_*i*_}, and the other observables, ${\cal M}_{k,{\mathrm{ini}}} \in \left\{ {M_{k,i_k}} \right\}$. (iii) In the third step, the system is driven out of equilibrium by steering its Hamiltonian in a time-dependent process, $\hat H \mapsto \hat H(t)$, for times 0 < *t* < *τ*. This defines a unitary time-evolution operator $\hat U(t)$ as the solution of $i\hbar \partial _t\hat U(t) = \hat H(t)\hat U(t)$, with $\hat U(0) = {\Bbb I}$, the identity operator on the system’s Hilbert space ${\cal H}$. Finally, (iv) at time *t* = *τ*, the system is projected on the eigenbasis of the instantaneous Hamiltonian, $\hat H\prime = \hat H(\tau )$. In general, the operators $\hat M_k$ will not commute with $\hat H(t)$ for *t* > 0, and we denote the set of charges that commute with $\hat H\prime$ as $\left\{ {\hat M_l ' } \right\}$(*l* = 1, …, ${\cal N}\prime$). Assuming that these operators commute with each other, this second projective measurement provides the quantities ${\cal E}_{{\mathrm{fin}}}\hskip -0.7pc \prime$ and $\left\{ {{\cal M}'_{l,{\mathrm{fin}}} } \right\}$, each belonging to the spectrum of the corresponding operator, in full analogy to the situation at *t* = 0. Thus, at the end of a single realisation of the FW process, one has collected the data set ${\cal D}_{{\mathrm{FW}}}$ = $\left\{ {{\cal E}_{{\mathrm{ini}}},\left\{ {{\cal M}_{k,{\mathrm{ini}}}} \right\};{\cal E}_{{\mathrm{fin}}}' ,\left\{ {{\cal M}_{l,{\mathrm{fin}}}' } \right\}} \right\}$ associated to the parameters $\vec \beta$ of the initial state Eq. (1).

The complementary BW process starts by preparing the system in an equilibrium state of the Hamiltonian $\hat H\prime$ (Fig. 1a). In accordance with the preceding discussion, this state will be of the GGE form with ${\cal N}\prime$ + 1 parameters, $\vec \beta \prime$ = $\left\{ {\beta ' ,\beta _1' , \ldots ,\beta _{{\cal N}\prime }' } \right\}$. At time *t* = 0, the system is projected on the basis $\left| {\vec f\prime } \right\rangle$ of simultaneous eigenvectors of $\hat H\prime$ and $\hat M_l^\prime$, obtaining the values ${\cal E}_{{\mathrm{ini}}}'$ and $\left\{ {{\cal M}_{l,{\mathrm{ini}}}' } \right\}$ for the corresponding observables. The system then evolves under the time-reversed protocol $\hat U^{ - 1}(t)$ for 0 < *t* < *τ*, so that at time *τ* its Hamiltonian is $\hat H$. A projective measurement on the final instantaneous eigenbasis provides values ${\cal E}_{{\mathrm{fin}}}$ and $\left\{ {{\cal M}_{k,{\mathrm{fin}}}} \right\}$ for the energy and other observables. A single realisation of the BW protocol thus gives a data set ${\cal D}_{{\mathrm{BW}}}$ = $\left\{ {{\cal E}_{{\mathrm{ini}}}' ,\left\{ {{\cal M}_{l,{\mathrm{ini}}}' } \right\};{\cal E}_{{\mathrm{fin}}},\left\{ {{\cal M}_{k,{\mathrm{fin}}}} \right\}} \right\}$ associated to the parameters $\vec \beta \prime$ of the BW initial state.

With the data sets ${\cal D}_{{\mathrm{FW}}}$ and ${\cal D}_{{\mathrm{BW}}}$ we build two (dimensionless) work-like quantities

2 $${\cal W}_{{\mathrm{FW}}} \equiv \beta {'}{\cal E}_{{\mathrm{fin}}}' + \mathop {\sum}\limits_{l = 1}^{{\cal N}\prime } {\kern 1pt} \beta _l' {\cal M}_{l,{\mathrm{fin}}}' - \left[ {\beta {\cal E}_{{\mathrm{ini}}} + \mathop {\sum}\limits_{k = 1}^{\cal N} {\kern 1pt} \beta _k{\kern 1pt} {\cal M}_{k,{\mathrm{ini}}}} \right],$$

3 $${\cal W}_{{\mathrm{BW}}} \equiv \beta {\cal E}_{{\mathrm{fin}}} + \mathop {\sum}\limits_{k = 1}^{\cal N} {\kern 1pt} \beta _k{\cal M}_{k,{\mathrm{fin}}} - \left[ {\beta {'}{\cal E}_{{\mathrm{ini}}}' + \mathop {\sum}\limits_{l = 1}^{{\cal N}\prime } {\kern 1pt} \beta _l' {\cal M}_{l,{\mathrm{ini}}}' } \right].$$

Due to the projective nature of the measurements, both these quantities are stochastic variables, and their statistics can be described through probability distribution functions (PDFs), ${\cal P}_{{\mathrm{FW}}}$ and ${\cal P}_{{\mathrm{BW}}}$, associated respectively to the FW and BW processes. We find that although the initial states of the two processes are independent, and may feature different numbers of charges and generalised inverse temperatures, these PDFs are not independent, but obey the following relation (see Methods):

4 $$\frac{{{\cal P}_{{\mathrm{FW}}}({\cal W})}}{{{\cal P}_{{\mathrm{BW}}}( - {\cal W})}}e^{ - {\cal W}} = \frac{{{\cal Z}' }}{{\cal Z}},$$

where ${\cal Z}\prime$ = ${\cal Z}\left( {\vec \beta \prime ,\hat H\prime ,\left\{ {\hat M_l^\prime } \right\}} \right)$ is the equilibrium partition function of the initial state of the BW process. By introducing (dimensionless) generalised free energy functions as ${\cal F}$ = −ln ${\cal Z}$ and ${\cal F}\prime$ = −ln ${\cal Z}\prime$, the right-hand side (r.h.s.) of Eq. (4) becomes exp(${\cal W}$ − Δ${\cal F}$), with Δ${\cal F}$ = ${\cal F}\prime$ − ${\cal F}$. Equation (4) is the generalisation of the TCR to systems with arbitrary numbers of charges associated to each equilibrium state, and to out-of-equilibrium processes that change the number of charges.

If we multiply both sides of Eq. (4) by ${\cal P}_{{\mathrm{BW}}}$(−${\cal W}$) and integrate over ${\cal W}$, we get

5 $$\left\langle {\left\langle {e^{ - {\cal W}}} \right\rangle } \right\rangle _{{\mathrm{FW}}} \equiv {\int}_{ - \infty }^\infty {\kern 1pt} {\mathrm{d}}{\cal W}{\kern 1pt} {\cal P}_{{\mathrm{FW}}}({\cal W})e^{ - {\cal W}} = e^{ - \Delta {\cal F}},$$

which is a generalisation of the QJE for systems with charges. Here we remark a qualitative difference between Eqs. (4) and (5). While the TCR relates the outcomes of driving processes starting in two different initial states, the QJE applies to a single system driven out of an equilibrium characterised by parameters $\vec \beta$. The apparent dependence on $\vec \beta \prime$ of Eq. (5) in fact shows that this equation relates an initial equilibrium state to all possible GGE-like states of the final Hamiltonian: for each possible set of ‘final’ equilibrium parameters $\vec \beta \prime$, the numerical values on the left-hand side (l.h.s.) and the r.h.s. of Eq. (5) will differ, but the equality will hold as long as the initial state is of the GGE form, Eq. (1). In this sense, the $\vec \beta \prime$ dependence in Eq. (5) is irrelevant, and one can test its validity taking, e.g., $\vec \beta \prime = \vec \beta$. This agrees with the intuition that by driving the system out of equilibrium we can obtain information on its initial equilibrium state (i.e., $\vec \beta$), but we cannot give physical meaning to the values of $\vec \beta \prime$ in Eq. (5). (The physics of Eqs. (4) and (5) is further discussed in Supplementary Note 2.)

More generally, consider the following scenario in which one of the constants of motion, say $\hat M_m$, commutes with the time-dependent Hamiltonian and with all the other charges at all times. Then, the time-dependent Hamiltonian can be set in a block-diagonal form, with different blocks corresponding to the different eigenvalues of this operator, and the values of $\hat M_m$ measured at the start and end of the TPM protocol must be identical. In this case, let us introduce a marginal generalised work ${\cal W}_m$ by ${\cal W}_m \equiv \beta {\prime}{\cal E}_{{\mathrm{fin}}}$ + $\mathop {\sum}\nolimits_{l \ne m} {\kern 1pt} \beta _l' {\cal M}_{l,{\mathrm{fin}}}'$ − $\left( {\beta {\cal E}_{{\mathrm{ini}}} + \mathop {\sum}\nolimits_{k \ne m} {\kern 1pt} \beta _k{\cal M}_{k,{\mathrm{ini}}}} \right)$. The corresponding PDF, ${\cal P}_{{\mathrm{FW}}}^{(m)}({\cal W}_m)$, satisfies a marginal version of the generalised TCR (see Methods):

6 $$\frac{{{\cal P}_{{\mathrm{FW}}}^{(m)}({\cal W}_m)}}{{{\cal P}_{{\mathrm{BW}}}^{(m)}( - {\cal W}_m)}}e^{ - {\cal W}_m} = e^{ - {\mathrm{\Delta }}{\cal F}}.$$

We show below how this result can be used to check whether a particular observable does or does not change in a non-equilibrium process without measuring it.

### Revealing missing charges

We now discuss how Eqs. (4)–(6) underlie novel strategies for two applications: (i) to reveal hidden conserved charges, and (ii) to check whether a particular observable of difficult experimental access does or does not change during a quantum non-equilibrium protocol. Next, we will illustrate this in practice through numerical studies of a trapped-ion quantum simulator.

The key realisation is that the r.h.s. of Eqs. (4)–(6) is determined solely by equilibrium properties pertaining to the initial states of the FW and BW processes, respectively, while the l.h.s. relates to measurement outcomes of non-equilibrium processes starting from those initial states, obtained via the TPM protocol. We can then check the completeness of the set of known charges by running the TPM protocol with a number, ${\cal N}_{{\mathrm{prot}}}$, of different protocols $\hat U$ (corresponding, for instance, to different durations *τ*). The experimental data corresponding to the ${\cal N}_{{\mathrm{prot}}}$ protocols provide different values for the l.h.s. of the generalised QJE, Eq. (5). As discussed above, these values depend physically on the ${\cal N}$ + 1 parameters $\vec \beta$ of the initial GGE state. The fact that all these expressions must equal the same single value on the r.h.s. entails a set of ${\cal N}_{{\mathrm{prot}}}$ (non-linear) equations for ${\cal N}$ + 1 unknowns. If we can find values for $\vec \beta$ that satisfy these equations, then we have accounted for all the charges required to describe the non-equilibrium dynamics of the system induced by $\hat U$; on the other hand, failure to find a satisfactory set of parameters points that more charges need to be included. A similar argument can be made based on the generalised TCR (4), to check whether all the charges in both FW and BW initial states have been accounted for. (Generally, the TCR depends on the ${\cal N}$ + ${\cal N}\prime$ + 2 parameters $\left\{ {\vec \beta ,\vec \beta \prime } \right\}$ characterising the FW and BW initial states. In practice, the number of required experiments can be notably reduced, e.g., by putting the system in contact with the same bath at the start of both FW and BW processes, so that $\vec \beta = \vec \beta \prime$, in which case only ${\cal N}$ + 1 unknowns need to be determined.) We note in addition that these completeness tests do not require prior knowledge of the inverse temperatures characterising the initial states; however, if we do have a reliable preliminary estimate of the inverse temperatures, this test allows to verify this estimate, or to conclude that the number of known charges is insufficient as soon as inconsistencies with Eqs. (4) and (5) emerge.

As a complement, Eq. (6) enables us to assess whether an operator that is known to commute with a Hamiltonian $\hat H$ does, or does not, change in a non-equilibrium procedure without measuring it, i.e., it enables to determine whether a non-equilibrium procedure transforms a charge into a dynamical variable. To see this, let us imagine that we are interested to know whether a certain observable of difficult experimental access, $\hat M_m$, is or not perturbed by a certain class of non-equilibrium protocols. To assess this, we perform a set of FW and BW protocols, excluding $\hat M_m$ from the TPM measurements, and calculate the marginal work ${\cal W}_m$. With these values, we build the associated PDFs, ${\cal P}_{{\mathrm{FW}}}^{(m)}$ and ${\cal P}_{{\mathrm{BW}}}^{(m)}$. If we can find values of $\vec \beta$ and $\vec \beta \prime$ without *β*_*m*_ such that the r.h.s. of Eq. (6) matches the data on the l.h.s., then the contributions of $\hat M_m$ to ${\cal F}$ and ${\cal F}\prime$ must cancel one another in Δ${\cal F}$, i.e., $\hat M_m$ has remained constant throughout the process. Otherwise, $\hat M_m$ cannot have the same value at the start and end of the protocol, i.e., $\hat M_m$ is a dynamical variable in the process.

### Application to a trapped-ion quantum simulator

Our generalised QFRs are valid for arbitrary unitary non-equilibrium processes, $\hat U$, applied to quantum systems with conserved quantities^16,29–31^. In the following, we illustrate their implications in the context of a trapped-ion experiment realisable with current technology^32–35^. First, we show that this system can be described by a Hamiltonian with a single charge. We then report numerical evidence showing how measurements of its work statistics in generic non-equilibrium protocols would violate the standard QJE and TCR—and how they agree with the predictions of our generalised QFRs. The fact that this model features a single charge makes it an especially attractive test ground, as this makes experimental tests of our generalised QFRs far more accessible than other models that would in principle require measurements on an infinite number of charges, such as the XXZ model.

We consider *N* ^43^Ca^+^ ions in an ion trap^32,33^ (Fig. 1b). Each ion can be described as a two-level system with internal states corresponding to two Zeeman levels within the ground ^2^*S*_1/2_ electronic state, whose energy splitting, *ħω*_at_, can be controlled by an external bias magnetic field^32,33^. The motional state of the ions in the trap is characterised by *N* − 1 collective modes in each direction^36^. Among these, we focus on the centre-of-mass (COM) mode, which couples identically to all ions and whose eigenfrequency, *ω*_COM_, is of the order of the trap’s oscillator frequency^36^. Internal and motional states can be coupled by light fields of frequency *ω* close to *ω*_±_ = *ω*_at_ ± *ω*_COM_, the blue (+) and red (−) motional sidebands. The Hamiltonian describing the dynamics of this system can be written in the form (see Methods and refs.^35,37–39^)

7 $$H{\mathrm{/}}\hbar = \omega _{{\mathrm{COM}}}\hat b^\dagger \hat b + \omega _{{\mathrm{at}}}\hat J_z \\ + \frac{{2g}}{{\sqrt N }}\left[ {(1 - \alpha )\left( {\hat J_ + \hat b + \hat J_ - \hat b^\dagger } \right) + \alpha \left( {\hat J_ + \hat b^\dagger + \hat J_ - \hat b} \right)} \right]$$

where $\hat b^\dagger$ and $\hat b$ are the operators creating and annihilating excitations in the COM mode, and $\hat J_s$ (*s* = *z*, +, −) are Schwinger spin operators describing the internal state of all the ions, with *J* = *N*/2. In Eq. (7) we have introduced *g* = (*Ω*_rsb_ + *Ω*_bsb_)/2 and *α* = *Ω*_bsb_/(*Ω*_rsb_ + *Ω*_bsb_), with *Ω*_bsb(rsb)_ the Rabi frequency characterising the coupling of internal and motional states through the first blue (red) motional sideband; these are functions of the light intensity at frequency *ω*_±_, respectively^35,36^. The Hamiltonian Eq. (7) is exactly that of the Dicke model^13,14^. For *α* = 0, it reduces to the Tavis–Cummings model, which is integrable and has an additional conserved quantity, $\hat M = \hat J + \hat J_z + \hat b^\dagger \hat b$ (see Methods and Supplementary Note 3). Thus, the dynamics of this system starting from an equilibrium state will be governed by our generalised QFRs. This can be verified by extending the filtering method^34,40^ used to verify the standard QJE^34^, to account for the internal structure of the ions in the Dicke model, so as to determine the initial and final energy values; indeed, as the spectrum of the Dicke model is non-degenerate, an energy measurement provides both ${\cal E}$ and ${\cal M}$. Additionally, the PDF of standard work can be obtained without projective measurements by utilising an ancilla qubit^41–43^.

In order to assess the existence of charges, we drive the system out of equilibrium. For simplicity, we consider a series of sudden quenches in space {*g*, *α*}; experimentally, these quenches correspond to changes in the intensities of the lasers realising the sideband couplings on a timescale much shorter than $\omega _{{\mathrm{COM}}}^{ - 1}$. We consider in particular the FW protocol {*g*_ini_, 0} → {*g*_int_, 1/2} → {*g*_fin_, 0}, with the system remaining in the intermediate stage for a variable time *τ* (Fig. 1c); this duration plays the role of the parameter *τ* characterising the ${\cal N}_{{\mathrm{prot}}}$ protocols in the procedure to reveal missing charges described above.

The choice *α* = 0 at *t* ∈ {0, *τ*} ensures that $\hat M$ commutes with both $\hat H$ and $\hat H\prime$. Hence, the initial equilibrium state of FW and BW processes will be of the GGE form with specific values for the inverse temperatures related to $\hat H$ and $\hat M$, which we label *β* and *β*_*M*_ (for concreteness, we analyse here the case that $\vec \beta \prime = \vec \beta$; see Supplementary Note 2 for a discussion on this choice). However, in the intermediate stages *α* = 1/2, which implies that there are no charges during an important part of the process. We will see that it is nevertheless possible to determine whether the system had a conserved quantity at the start of the process. (Additional simulations in Supplementary Note 4 for a process ending in *α* = 1/2, i.e., where $\hat M$ and $\hat H\prime$ do not commute, support analogous conclusions, see Supplementary Fig. 1.)

**Fig. 2:**
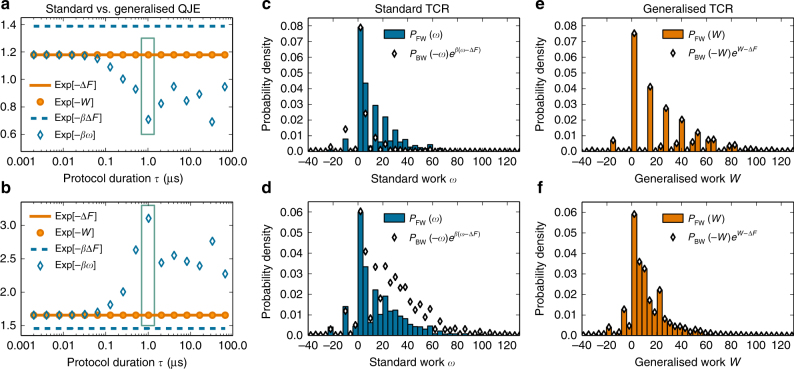
Generalised quantum fluctuation relations. **a** Plot of exp (−Δ${\cal F}$) (solid orange line) and exp(−*β*Δ${\cal F}$) (dashed blue line), compared with the exponentiated averages $\left\langle {\left\langle {{\mathrm{exp}}( - {\cal W})} \right\rangle } \right\rangle$ (circles) and $\left\langle {\left\langle {{\mathrm{exp}}( - \beta w)} \right\rangle } \right\rangle$ (open diamonds) for protocols with duration *τ* ∈ [1 ns, 100 μs] that start in a GGE state given by (*βε*_0_ = 0.1, *β*_*M*_*ε*_0_ = 0.3). **b** Same as **a** with *β*_*M*_*ε*_0_ = −0.1. **c** Probability distribution function (PDF) of standard work for the FW process, *P*_FW_(*w*) (blue bars), and PDF for the BW process weighted according to the standard TCR, exp[*β*(*w* − Δ*F*)]*P*_BW_(−*w*) (diamonds) for the process with duration *τ* = 1.024 μs [green box in **a**]. **d** Same as **c** for the process indicated in **b**. **e**, **f** PDF of generalised work for the FW process, ${\cal P}_{{\mathrm{FW}}}$(${\cal W}$) (orange bars), and PDF of generalised work for the BW process weighted according to the generalised TCR exp (${\cal W}$ − Δ${\cal F}$)${\cal P}_{{\mathrm{BW}}}$(−${\cal W}$) (diamonds) for the process indicated by a box in **a** and **b**, respectively. Parameters for all simulations: *N* = 7 ions, *ω*_COM_ = 3*ε*_0_, *ω*_at_ = 10*ε*_0_, *g*_ini_ = 2*ε*_0_, *g*_int_ = 3*ε*_0_ and *g*_fin_ = *ε*_0_, with *ε*_0_ = *ħ* × 2*π* MHz a typical value trapping energy scale in trapped-ion experiments^32–35^

We show in Fig. 2a the average exponentiated work performed on the system by a FW process as a function of duration *τ*. We compare the results using standard work, $\left\langle {\left\langle {{\mathrm{exp}}\left( { - \beta _w} \right)} \right\rangle } \right\rangle _{{\mathrm{FW}}}$, to those with generalised work $\left\langle {\left\langle {{\mathrm{exp}}\left( { - {\cal W}} \right)} \right\rangle } \right\rangle _{{\mathrm{FW}}}$. We see that the standard work average varies by up to 40% for durations *τ* ≳ 0.1 μs. This *τ* dependence indicates that the standard work is no longer the relevant magnitude in non-equilibrium processes: there is one (or more) missing charge(s) whose fluctuations need to be taken into account to describe the measurement outcomes. In contrast to this, the average of generalised work including $\hat M$ remains constant for all *τ*; it thus follows from Eq. (5) that this definition of ${\cal W}$ includes all the relevant charges of the system.

Notably, the two averages agree with each other for short processes, *τ* < 0.1 μs. This reflects that, at short times, $\hat M$ remains approximately constant and a marginal TCR is expected to hold. Importantly, however, both averages agree with the prediction of the generalised QFRs, i.e., exp(−Δ${\cal F}$), while the expectation that excludes $\beta _M\hat M$ (dashed line) is off by 20%. In practice, this means that a nave fit of the $\left\langle {\left\langle {{\mathrm{exp}}\left( { - \beta _w} \right)} \right\rangle } \right\rangle$ data to exp (−*β*Δ*F*) would yield a biased value for *β*, i.e., a wrong estimate of the initial temperature of the system. Generally, use of the standard QFRs will provide biased estimates if the system has charges. Figure 2b support analogous conclusions for the case *β*_*M*_ < 0.

The relevance of charge fluctuations in the non-equilibrium dynamics becomes apparent in Fig. 2c–f, which portray the statistics of standard and generalised work under driving protocols with *τ* = 1 μs. In Fig. 2c, d we observe that scaling the PDF of standard work for the BW process by exp[*β*(*w* − Δ*F*)] (diamonds) does not agree with the PDF of the FW process (bars), in contrast to the prediction of the standard TCR. Such a disagreement is a strong indication of the existence of charges in a system. On the other hand, the prediction of the generalised TCR, Eq. (4), is satisfied with great accuracy for both *β*_*M*_ > 0 [Fig. 2e] and *β*_*M*_ < 0 (Fig. 2f). In other words, the TCR equality is only fulfilled when all relevant charges are included in the calculations of ${\cal W}$ and Δ${\cal F}$, and a discrepancy between the measurable quantities ${\cal P}_{{\mathrm{FW}}}$(${\cal W}$) and ${\cal P}_{{\mathrm{BW}}}$(${\cal W}$) exp(${\cal W}$ − Δ${\cal F}$) points to the existence of hidden charges that need to be included.

## Discussion

Our findings extend the foundations of quantum thermodynamics with charges^44–48^ to non-equilibrium processes with various (generalised) baths and/or that break one or more conservation laws, while they revert to the standard QFRs in the case that the Hamiltonian is the only conserved quantity (${\cal N}$ = ${\cal N}\prime$ = 0) and both processes start at the same inverse temperature, *β*′ = *β*. In this case, ${\cal W}_{{\mathrm{BW}}}$ = −${\cal W}_{{\mathrm{FW}}}$ = −*βw* and Eq. (4) becomes the standard TCR^21^ with the standard free energy *F* = −*β*^−1^ ln ${\cal Z}$. In the same conditions, Eq. (5) recovers the standard QJE^22–24^. In addition, if ${\cal N}$ = ${\cal N}\prime$ and $\vec \beta = \vec \beta \prime$, Eq. (4) reduces to the version of the TCR for GGEs derived in ref. ^44^ under the assumption of continuity of the charges during the driving protocols.

Our numerical results in Fig. 2a–d highlight the limitations of the standard QFRs in dealing with systems with charges, in particular in order to extract equilibrium properties by means of non-equilibrium measurements^41–43,49,50^. Our generalised QFRs, Eqs. (4) and (5), provide the robust theoretical basis necessary to do that, as evidenced by the exquisite agreement of our numerical simulations with their predictions (Fig. 2a, b, e, f).

The framework informed by the approach based on our QFRs is of general applicability to quantum many-body systems. In particular, our protocol to reveal the existence of charges enables one to uncover more general charges than those related to transport properties and governed by the Mazur bound^17–20,51^, as illustrated by our analysis of the Dicke model. Ongoing developments in several physical platforms—e.g., trapped ions^32–35^ and superconducting circuits^52,53^—readily enable precise experimental investigations of this model, which will further inform the development of tools to study non-equilibrium quantum dynamics, and its exploitation in practical tasks^54,55^. We remark as well the possibility of simultaneously revealing new charges and determining the associated (equilibrium) inverse temperatures borne in our protocol.

An important question that remains open is how to determine the exact form of the charge operators. Indeed, their intimate dependence on the associated Hamiltonian makes it hard to give a general prescription. Still, we conjecture that a strategy based on analysing the behaviour of different candidate operators under various driving protocols by means of the marginal TCR, Eq. (6), will illuminate the exact form of the charges. This possibility, which lies beyond the scope of this work, will be explored in the future.

These findings will be relevant to fundamental studies on relaxation and thermalisation of quantum systems^56–58^, and to the advance of quantum simulation and quantum probing protocols that exploit QFRs^41–43,49,50^. Our results extend the foundations of quantum thermodynamics with charges^44–48^ to non-equilibrium processes with various (generalised) baths and/or that break one or more conservation laws, while they revert to the standard QFRs in the case that the Hamiltonian is the only conserved quantity (${\cal N}$ = ${\cal N}\prime$ = 0) and both processes start at the same inverse temperature, *β*′ = *β*. In this case, ${\cal W}_{{\mathrm{BW}}}$ = −${\cal W}_{{\mathrm{FW}}}$ = −*βw* and Eq. (4) becomes the standard TCR^21^ with the standard free energy *F* = −*β*^−1^ ln ${\cal Z}$. In the same conditions, Eq. (5) recovers the standard QJE^22–24^. In addition, if ${\cal N}$ = ${\cal N}\prime$ and $\vec \beta = \vec \beta \prime$, Eq. (4) reduces to the version of the TCR for GGEs derived in ref. ^44^ under the assumption of continuity of the charges during the driving protocols.

We further remark that our QFRs do not assume any particular relationship between the sets of observables that commute with $\hat H$ and $\hat H\prime$; in particular, we do not assume continuity between these sets^44^; this is important, as frequently even a small perturbation of a Hamiltonian transforms its charges into dynamical quantities. Thus, our generalised QFRs enable us to study a larger class of non-equilibrium processes, such as cyclic processes that include an intermediate thermalisation step where the system remains isolated and thus equilibrates to a GGE whose generalised temperatures are not fixed beforehand (see Supplementary Note 2 for more details). This opens the door to studying the thermalisation of an integrable system when perturbed away from integrability^16,30,59^. We thus expect our work will contribute to illuminate open fundamental questions on thermalisation, localisation and ergodicity, especially in the presence of integrals of motion^60–64^. Besides, due to the enhanced role of fluctuations in small systems, we expect our work will contribute to a better understanding and improved design of new micro- and nanometre-sized devices where the interplay of thermal and quantum effects is paramount^54,65,66^, and thus to address questions related to cyclic protocols and the efficiency of quantum heat engines^54,55^, thermometry of strongly correlated systems at ultra-low temperatures^49,67^ and novel quantum sensing applications based on quantum information theory and quantum thermodynamics^50,68,69^.

In summary, we have derived a set of generalised QFRs relevant to unitary non-equilibrium processes starting from states of the GGE, which correspond to equilibrium states of quantum system with charges. Based on these generalised QFRs, we have proposed a general method to address the question of identifying all charges relevant to the non-equilibrium dynamics of a quantum system^17–19,51^. We provided robust numerical evidence of the measurable impact that these theoretical findings can have in current studies with quantum simulators; in particular, we highlighted the importance of identifying all conserved charges to obtain unbiased estimates of their equilibrium properties, such as the temperature, through non-equilibrium measurements^41–43,49^. In addition, we put forward a scheme to determine whether an integral of motion is affected by a class of non-equilibrium processes, without measuring it.

## Methods

### Derivation of the generalised QFRs

To derive the TCR (4), let us introduce the shorthand notations ${\cal A}_{\vec i} = \beta E_{i_0} + \mathop {\sum}\nolimits_k {\kern 1pt} \beta _kM_{k,i_k}$ and ${\cal A}_{\vec f}^\prime = \beta \prime E_{f_0}^\prime + \mathop {\sum}\nolimits_l {\kern 1pt} \beta _l' M_{l,f_l}'$, with $\left| {\vec i} \right\rangle$ = $\left| {i_0, \ldots ,i_{\cal N},\eta } \right\rangle$ and $\left| {\vec f\prime } \right\rangle$ = $\left| {f_0, \ldots ,i_{{\cal N}\prime },\eta \prime } \right\rangle$. Then, the probability that a realisation of the protocol requires an amount ${\cal W}$ of generalised work (2) reads

8 $${\cal P}_{{\mathrm{FW}}}({\cal W}) \equiv {\sum} _{\vec i,\vec f}{\kern 1pt} p_{\vec i}\pi _{\vec i \to \vec f}(\hat U)\delta \left[ {{\cal W} - \left( {{\cal A}\prime - {\cal A}} \right)} \right],$$

Here $p_{\vec i} = {\mathrm{exp}}\left( { - {\cal A}_{\vec i}} \right){\mathrm{/}}{\cal Z}$, ${\cal Z} = \mathop {\sum}\nolimits_{\vec i} {\kern 1pt} {\mathrm{exp}}\left( { - {\cal A}_{\vec i}} \right)$, is the probability to find the system in state $\left| {\vec i} \right\rangle$ in the first projective measurement at *t* = 0, and $\pi _{\vec i \to \vec f}\left( {\hat U} \right) = \left| {\left\langle {\vec f\prime } \right|\hat U\left| {\vec i} \right\rangle } \right|^2$ is the probability that the system initially in state $\left| {\vec i} \right\rangle$ is found in state $\left| {\vec f\prime } \right\rangle$ after the protocol $\hat U$; finally, *δ*(0) = 1 and otherwise *δ*(*x*) = 0. The PDF (8) can be rewritten as

9 $$\begin{array}{l}{\cal P}_{{\mathrm{FW}}}({\cal W}) = \\ = {\sum} _{\vec i,\vec f}{\kern 1pt} \frac{{{\mathrm{exp}}\left( { - {\cal A}_{\vec i}} \right)}}{{\cal Z}}\left| {\left\langle {\vec f\prime } \right|U\left| {\vec i} \right\rangle } \right|^2\delta \left[ {{\cal W} - \left( {{\cal A}_{\vec f}^\prime - {\cal A}_{\vec i}} \right)} \right]\\ = {\sum} _{\vec i,\vec f}{\kern 1pt} \frac{{{\mathrm{exp}}\left( {{\cal W} - {\cal A}_{\vec f}^\prime } \right)}}{{\cal Z}}\left| {\left\langle {\vec f\prime } \right|U\left| {\vec i} \right\rangle } \right|^2\delta \left[ {{\cal W} - \left( {{\cal A}_{\vec f}^\prime - {\cal A}_{\vec i}} \right)} \right]\\ = \frac{{{\cal Z}\prime }}{{\cal Z}}{\sum} _{\vec i,\vec f}{\kern 1pt} \frac{{{\mathrm{exp}}\left( {{\cal W} - {\cal A}_{\vec f}^\prime } \right)}}{{{\cal Z}\prime }}\left| {\left\langle {\vec i} \right|U^{ - 1}\left| {\vec f\prime } \right\rangle } \right|^2\delta \left[ {{\cal W} + \left( {{\cal A}_{\vec i} - {\cal A}_{\vec f}^\prime } \right)} \right]\\ \equiv \frac{{{\cal Z}\prime }}{{\cal Z}}e^{\cal W}{\cal P}_{{\mathrm{BW}}}( - {\cal W}).\end{array}$$

In the first step we substituted $p_{\vec i}$ and $\pi _{\vec i \to \vec f}$, in the third step we applied that $\hat U$ is unitary and in the last step we identified the PDF corresponding to the time-reversed process. This completes the proof of Eq. (4), from which the generalised QJE (5) follows as discussed in the main text. The PDFs of standard (dimensionful) work, $w = {\cal E}_{{\mathrm{fin}}}^\prime - {\cal E}_{{\mathrm{ini}}}$, shown in Fig. 2, are defined analogously, e.g., $P_{{\mathrm{FW}}}(w)$ = $\mathop {\sum}\nolimits_{\vec i,\vec f} {\kern 1pt} p_{\vec i}\pi _{\vec i \to \vec f}\delta \left[ {w - \left( {{\cal E}_{{\mathrm{fin}}}^\prime - {\cal E}_{{\mathrm{ini}}}} \right)} \right]$, which follows from the standard situation with the same equilibrium conditions at the start of FW and BW processes, *β*′ = *β*. Finally, the marginal TCR (6) is derived in a manner analogous to (9) by defining the PDF of marginal work as ${\cal P}_{{\mathrm{FW}}}^{(m)}\left( {{\cal W}_m = x} \right)$ ≡ $\mathop {\sum}\nolimits_{\vec i,\vec f} {\kern 1pt} p_{\vec i}\pi _{\vec i \to \vec f}\delta \left[ {x - \left( {{\cal A}_f^{\prime (m)} - {\cal A}_{\vec i}^{(m)}} \right)} \right]$ with ${\cal A}_{\vec i}^{(m)}$ = $\beta {\cal E}_{{\mathrm{ini}}} + \mathop {\sum}\nolimits_{k \ne m}^N {\kern 1pt} \beta _k{\cal M}_{k,{\mathrm{ini}}}$ and ${\cal A}_{\vec f}^{\prime (m)}$ = $\beta \prime {\cal E}_{{\mathrm{fin}}}^\prime + \mathop {\sum}\nolimits_{l \ne m}^{{\cal N}\prime } {\kern 1pt} \beta _l^\prime M_{l,{\mathrm{fin}}}^\prime$.

We remark that the fundamental assumptions underlying our generalised QFRs are (i) that the state of the system at the start of both FW and BW processes is of the GGE form, and (ii) the the corresponding driving protocols are time-reversed of each other.

### Dicke model with trapped ions

Consider *N* ions in an ion trap^36^. Each ion can be described as a two-level system (qubit) with internal states $\left\{ {\left| \uparrow \right\rangle ,\left| \downarrow \right\rangle } \right\}$ corresponding to two Zeeman levels within the ground ^2^*S*_1/2_ electronic state. Their energy splitting can be controlled by an external magnetic field, *B*, as *ħω*_at_ = Δ*μB*, where Δ*μ* is the difference in magnetic moments of the two internal states^32, 33^. The motional state of the ions in the trap is characterised by *N* − 1 collective modes in each direction^36^. Among these, the COM mode, with eigenfrequency *ω*_COM_, is characterised by coupling identically to all ions. Internal and motional states can be coupled by radiation of frequency *ω* close to *ω*_at_ ± *ω*_COM_, the blue (red) motional sideband^36^.

The complete Hamiltonian describing the dynamics of the system reads *H* = *H*_0_ + *H*_JC_ + *H*_aJC_ with *H*_0_ = $\hbar \omega _{{\mathrm{COM}}}\left( {\hat b^\dagger \hat b + 1{\mathrm{/}}2} \right)$ + $\mathop {\sum}\nolimits_{l = 1}^N {\kern 1pt} \hbar \omega _{{\mathrm{at}}}\sigma _z^{(l)}$, where $\hat b^\dagger$ and $\hat b$ are the operators creating and annihilating excitations (phonons) in the COM mode and $\sigma _z^{(l)}$ = $\left| \uparrow \right\rangle _{(l)}\left\langle \uparrow \right| - \left| \downarrow \right\rangle _{(l)}\left\langle \downarrow \right|$ is the Pauli *z* operator for ion *l* = 1, …, *N*. The coupling between the ions’ internal state and the COM mode mediated by radiation is given by the Jaynes–Cummings (JC) and anti-JC Hamiltonians, *H*_JC_ = $\mathop {\sum}\nolimits_{l = 1}^N \left( {\hat b\sigma _ + ^{(l)} + \hat b^\dagger \sigma _ - ^{(l)}} \right)\hbar { {\Omega }}_{{\mathrm{rsb}}}{\mathrm{/}}2$ and *H*_aJC_ = $\mathop {\sum}\nolimits_{l = 1}^N \left( {\hat b^\dagger \sigma _ + ^{(l)} + \hat b\sigma _ - ^{(l)}} \right)\hbar { {\Omega }}_{{\mathrm{bsb}}}{\mathrm{/}}2$, with the raising (lowering) operators $\sigma _ + ^{(l)} = \left| \uparrow \right\rangle _{(l)}\left\langle \downarrow \right|$ and $\sigma _ - ^{(l)} = \left[ {\sigma _ + ^{(l)}} \right]^\dagger$. *Ω*_bsb(rsb)_ is the Rabi frequency of the blue (red) motional sideband^36^.

The internal quantum state of a single ion can be mapped onto an effective spin-1/2 system. The full quantum state of the ions in the trap can then be expressed in the basis $\left| {J,J_z,n} \right\rangle$ = $\left| {J,J_z} \right\rangle \otimes \left| n \right\rangle$, where $\left| n \right\rangle$ is the Fock state with *n* = 0, 1, 2,… excitations in the COM mode, and $\left| {J,J_z} \right\rangle$ is an eigenstate of the collective Schwinger pseudo-spins $\hat J_s = \mathop {\sum}\nolimits_{l = 1}^N {\kern 1pt} \sigma _s^{(l)}$ (*s* = *z*, +, −), with *J* = *N*/2 and *J*_*z*_ = −*J*, −*J* + 1, …, *J*. Using the collective pseudo-spins and dropping constant terms, the Hamiltonian *H* can be conveniently rewritten as Eq. (7), with the coupling parameters given in terms of the Rabi frequencies within *H*_JC_ and *H*_aJC_ by *g* = (*Ω*_rsb_ + *Ω*_bsb_)/2 and *α* = *Ω*_bsb_/(*Ω*_rsb_ + *Ω*_bsb_).

### Numerical calculations

We solve the time evolution of the system with *N* = 7 ions by exact propagation with the full interacting Hamiltonian expressed in the basis of eigenstates $\left| {J,J_z,n} \right\rangle$ with *J* = 7/2, *J*_*z*_ = −7/2, …, 7/2, and *n* = 0, 1, …, *n*_max_. We have verified that a maximum phonon occupation *n*_max_ = 800 is sufficient to faithfully simulate the evolution for the timescales of interest. To simulate the TPM protocol, we proceed in the following way. We start the process in a given eigenstate of the system, with definite eigenvalues of the Hamiltonian, $\hat H$, and the conserved charge, $\hat M$, $\left| {E_n,M_m} \right\rangle$. Then, we perform a sudden quench to the intermediate stage and, then, another quench to the final Hamiltonian. We calculate the probability of each transition $\left| {E_n,M_m} \right\rangle$ → $\left| {E_p^\prime ,M_r^\prime } \right\rangle$, involving a work $w = E_p^\prime - E_n$ and a change in the conserved charge, $w_M = M_r^\prime - M_m$; this probability is $P\left( {w,w_M} \right)$ = $\left| {\left\langle {E_p^\prime ,M_r^\prime |E_n,M_m} \right\rangle } \right|^2$. From this result, we obtain the marginal probabilities for the work, *w*, and the change in the charge, *w*_*M*_; both values provide us the generalised work required by the transition, Eqs. (2) and (3)^44–46^. We repeat the same calculations for every eigenstate of the initial system, obtaining the corresponding marginal probability distributions. The final results follow by averaging the different initial states with the probability distribution given by the GGE, with the corresponding temperatures *β* and *β*_*M*_. Note that this procedure is totally equivalent to averaging over a large number of realisations consisting in: first selecting randomly an initial eigenstate $\left| {E_n,M_m} \right\rangle$, with the probability distribution given by the GGE (simulating the first projective measurement); and second, selecting randomly the final state as an eigenstate of the final Hamiltonian, with a probability distribution given by $\left| {\left\langle {E_p^\prime ,M_r^\prime |E_n,M_m} \right\rangle } \right|^2$ (simulating the second projective measurement). Moreover, this numerical procedure is in direct analogy with the implementation of the filtering method in ref. ^34^ to project the initial state onto a given eigenstate of the system.

In all the simulations shown, we use *g*(*t* < 0) = 2*ε*_0_, *g*(0 < *t* < *τ*) = 3*ε*_0_ and *g*(*τ*) = *ε*_0_. This choice entails that the coupling constant in the intermediate stage is above the critical coupling, *g*_cr_ = $\sqrt {\omega _{{\mathrm{COM}}}{\kern 1pt} \omega _{{\mathrm{at}}}} {\mathrm{/}}2\sim 2.74\varepsilon _0$, for the transition from normal to super-radiant behaviour of the Dicke model (see Supplementary Note 3). Indeed, we have checked that the majority of the populated levels at the end of the protocol lays in the chaotic regime. Thus, we expect an effective breakdown of the conservation of $\hat M_{}^{}$, and a complete thermalisation for sufficiently large *τ*^70^.

### Data availability

The data that support the findings of this study are available from the corresponding author on request.

## Electronic supplementary material

Supplementary Information
