# Supplementary material for: Revealing missing charges with generalised quantum fluctuation relations
Source: Nat Commun. 2018 May 22;9:2006. doi: 10.1038/s41467-018-04407-1 (PMC5964258; doi:10.1038/s41467-018-04407-1)
Supplement: Supplementary file 1 — Supplementary Information [file 41467_2018_4407_MOESM1_ESM.pdf]

Supplementary Information:
Revealing missing charges with generalised quantum fluctuation relations

J. Mur-Petit et al.

Supplementary Note 1
Equilibration and thermalisation in systems with charges

The main result of our paper is the derivation of a set of quantum fluctuation relations that apply to non-equilibrium processes in system whose initial state is of the form of a GGE density matrix (Eq. (1)in main text). Such a density matrix describes an equilibrium thermal state for a system with charges, and so it is important to discuss if and when one can expect a quantum system to be able to relax to such a state, at least in the sense that expectation values calculated with a GGE density matrix constitute reliable predictions of the system's physical properties. The study of equilibration and thermalisation in isolated quantum systems is a very active research field, both theoretically and experimentally. In this Supplementary Discussion we review its most relevant results and how they relate to the main part of our work.

As in the main text, we will be concerned with the evolution of a quantum system starting from a certain initial state —which we do not require now to be an equilibrium state— in absence of external perturbations. If the initial state is pure, $|\psi(0)\rangle$, the time-evolved state remains always pure, $|\psi(t)\rangle$, and therefore nothing like a relaxation to a (mixed) equilibrium state occurs. However, under almost any circumstances, the density matrix corresponding to the actual time-evolved state, $\rho(t) = |\psi(t)\rangle\langle\psi(t)|$, remains almost always close to an effective equilibrium state $\rho_{\text{eq}} = \lim_{T\to\infty}(1/T)\int_0^T dt\,\rho(t)$: the amount of time during which $\rho(t)$ is far from ρ_{eq} is negligible [1]. As a consequence, the long-time average of any physical observable $\hat{\mathcal{O}}$ coincides with the statistical average over the former effective equilibrium state, $\langle\hat{\mathcal{O}}\rangle_{\text{eq}} = \text{Tr}\left[\rho_{\text{eq}}\hat{\mathcal{O}}\right]$. This result is mathematically proven under very general assumptions [2–6], so it is expected to hold in any experiment. Unfortunately, the proof says nothing about the shape of ρ_{eq}; in particular, it does not link ρ_{eq} with the usual statistical ensembles, like the Generalised Gibbs Ensemble, ρ_{GGE}. Furthermore, it is straightforward to see that, if the spectrum of the system is non-degenerate, $E_m \neq E_n$, $\forall n \neq m$, this equilibrium state reads,

$$\rho_{\text{eq}} = \sum_n |C_n|^2 |n\rangle\langle n|, \tag{1}$$

where $|n\rangle$ represents the eigenstate of the system with energy E_n; the coefficients C_n depend on the initial state, $C_n = \langle\psi(0)|n\rangle$, and $|\psi(0)\rangle$ is the initial state itself. In other words, *the equilibrium state stores a lot of information about the initial state*, encoded in the coefficients $|C_n|^2$. This fact is compatible neither with standard thermodynamics (according to which the expectation value of the energy, E, is enough to properly describe the equilibrium state), nor with the predictions of the GGE (which establishes that the equilibrium state depends on the expectation values of a few macroscopic observables: a small number of conserved charges, $\hat{M}_k$). Indeed, Supplementary Eq. (1) is compatible with GGE only if the number of conserved charges is equal to the dimension of the phase space, and $\hat{M}_k = |k\rangle\langle k|$.

This paradox is solved by the eigenstate thermalisation hypothesis (ETH), at least in highly chaotic quantum systems [7, 8]. In this kind of systems, expectation values of *physical observables*, $\hat{\mathcal{O}}$, change smoothly with energy, i.e.,

$$\begin{aligned}\mathcal{O}_{n,n} &\equiv \langle n|\hat{\mathcal{O}}|n\rangle = f(E)\\ &\approx f(E_n) + \frac{1}{2}\frac{\partial^2 f}{\partial E^2}(E_n - E)^2 + O(E_n - E)^3\end{aligned} \tag{2}$$

with the linear term vanishing by symmetry [8–12]. As a consequence, the expectation values of such observables in the equilibrium ensemble read

$$\langle\hat{\mathcal{O}}\rangle_{\text{eq}} = \sum_n |C_n|^2\,\mathcal{O}_{n,n} = \sum_n |C_n|^2 f(E_n). \tag{3}$$

Let us now consider an initial state for which the values of the coefficients $|C_n|^2$ are peaked around an energy E, with a width σ_E characterising the spread of the distribution. If the distribution is 'narrow', i.e., $\sigma_E^2\left(\partial^2 f/\partial E^2\right) \ll f(E)$, we can safely say that

$$\langle\hat{\mathcal{O}}\rangle_{\text{eq}} = \sum_n |C_n|^2 f(E) = f(E), \tag{4}$$

independently of the actual values of the coefficients C_n [13]. In other words, according to the ETH, the expectation value $\langle\hat{\mathcal{O}}\rangle$ is the same over any sufficiently narrow distribution. Hence, highly chaotic quantum systems are expected to thermalise independently of the initial condition, $|\psi(0)\rangle$, at least for *physical observables.*

Two important caveats concern this result. First, there is not a clear definition of *physical observables* to which Supplementary Eq. (4) applies. For example, it is quite obvious that projectors over the eigenstates, $\hat{\mathcal{O}}_j = |j\rangle\langle j|$, do not satisfy the conditions of the ETH. Furthermore, the condition $\sigma_E^2\left(\partial^2 f/\partial E^2\right) \ll f(E)$ strongly depends on the shape of the observable; one can easily find operators $\hat{\mathcal{O}}$ not satisfying it given a particular width of the energy distribution, σ_E^2. Second, if a quantum system has a number of charges, the ETH does not hold either. On the contrary, the expectation value of an observable in a particular eigenstate largely depends on the quantum numbers determined by the charges. So, if two consecutive energy levels, say $|n\rangle$ and $|n+1\rangle$, have different values of these quantum numbers, we can generically expect that $\mathcal{O}_{n,n}$ to be notably different from $\mathcal{O}_{n+1,n+1}$.

The GGE is introduced to solve the second caveat. If a quantum system has a number of relevant charges, it is conjectured that a GGE density matrix properly describes the expectation values of *physical* observables in the equilibrium states. Unfortunately, as discussed in the main text, it is not easy to establish how many

charges are required to build the GGE for a generic system. (As we have pointed above, if this number equals the dimension of the Hilbert space, the corresponding GGE exactly reproduces the equilibrium state $\rho_{\rm eq}$.) It is possible to find certain initial conditions for which a small number of charges is enough to properly describe equilibrium states, whereas the actual time-evolved state fluctuates around a totally different equilibrium state, if the experiment starts from another initial state. A generalisation to the ETH for these systems, called generalised eigenstate thermalisation hypothesis, has been proposed, but it is not based in so solid grounds as the ETH [14]. In fact, an important number of exceptions to its predictions for the XXZ model were reported (see e.g. [15–17]). While these particular difficulties with the XXZ model have been nowadays resolved by the the identification of relevant quasi-local charges [18–21], this episode points to the limitations of the generalised ETH.

Summarising, even though there exists a mechanism leading to thermalisation in highly chaotic systems, it does apply neither to all kind of observables, nor to systems with conserved charges. So, the choice of the observables used to test whether a quantum system is properly thermalised, or if all the relevant conserved charges are taken into account, is a tricky task that can lead to misleading conclusions. In this work we propose an alternative method based in generalised quantum fluctuation theorems, not depending on particular observables.

Supplementary Note 2
Physical content of the QFRs

In the main part of this paper, we have derived new QFRs that relate the fluctuations when a quantum systems is subject to two non-equilibrium processes (labelled 'forward' and 'backward') starting from equilibrium states with a (possibly) different number of charges, and (possibly) different generalised temperatures, $\vec{\beta} \neq \vec{\beta}'$. As standard QFRs are concerned with the situation that the initial state of the FW and BW processes is at the same inverse temperature, we clarify here the physical content derived from considering this more general situation.

The link that Eqs. (4) and (5) in the main text establish between different GGE states builds on the definitions of the quantities $\mathcal{W}_{\rm FW}, \mathcal{W}_{\rm BW}$. Physically, we can associate a state function to each GGE state; e.g., for the state at the start of the FW process, we define $\mathcal{A}(\vec{\beta}) = \beta\langle\hat{H}\rangle + \sum_k \beta_k \langle\hat{M}_k\rangle$, with $\langle\cdots\rangle = {\rm Tr}[\hat{\rho}_{\rm GGE}(\vec{\beta})\cdots]$, cf. [22, 23]. It is straightforward to see that in absence of charges, $\mathcal{A}$ reduces to the system's energy multiplied by its inverse temperature. The quantity $\mathcal{W}_{\rm FW}$ (respectively, $\mathcal{W}_{\rm BW}$) then measures how much this state function changes as the process $\hat{U}$ (resp. $\hat{U}^{-1}$) takes the system away from the initial state, much as standard work measures how much energy is pumped into the system in a non-equilibrium process. Generally the system at the end of each process is out of equilibrium, and it is not possible to associate to it a definite value of a state function. However, the microscopic reversibility of the laws of physics, embodied here in the unitarity of $\hat{U}$, allows to establish robust connections, in the form of Eqs. (4) and (5), between the out-of-equilibrium fluctuations of the energy and charges of an equilibrium state of $\hat{H}$ with those of any equilibrium state of $\hat{H}'$.

TCR and cyclic processes

As the TCR measures the statistical asymmetry in the work PDFs of the FW and BW processes, an exact knowledge of both $\vec{\beta}$ and $\vec{\beta}'$ is required. The standard TCR and its generalisation in [24] are derived assuming equal baths at the start of both processes. Our formalism makes it possible instead to study more general processes, including:

(i) Processes in which the system is connected to equal baths before the forward and the backward parts. They constitute the natural generalisation of the standard TCR; the number of conserved charges and the corresponding temperatures are the same in both initial states. Hence, the physical interpretation of the generalised TCR is straightforward. The mechanical generalised work required to complete the forward part of the process can be defined as

$$W \equiv \mathcal{W}/\beta = w + \sum_{k=1}^{\mathcal{N}} \frac{\beta_k}{\beta} \Delta M_k, \tag{5}$$

where w is the mechanical work. So, W can be understood as a generalised mechanical energy. If the process is done slowly enough, $W = \Delta\mathcal{F}/\beta$, and all of these generalised energy can be recovered in the backward part of the process. Otherwise, a part of the generalised mechanical energy will be dissipated into the final baths by a flux of generalised heat, $\mathcal{Q} = W - \Delta\mathcal{F}/\beta$, and this flux is linked to the entropy produced by the process, $\Delta S = \beta Q$ (we take $k_B = 1$). After the backward part of the process, when the cycle is completed, $\Delta\mathcal{F} = 0$, and the entropy produced is sent to the rest of the universe.

(ii) Processes in which the system is connected to different baths before the forward and the backward parts, including the case of baths that can have a different number of charges. In this case, the interpretation in terms of the flux of generalised heat and the increase of entropy is not so clean, since there is no single temperature β. However, the magnitude $\mathcal{W} - \Delta\mathcal{F}$ still represents a measure of the irreversibility of the process. Exactly as in the former case, when the cycle is completed $\Delta\mathcal{F} = 0$, and the entropy produced is sent to the rest of the universe.

(iii) Processes completed in isolation. In this case, the system starts at a particular initial state, given

by the temperatures β_k, and relaxes into another particular state after the forward part of the process, characterised by a new set of temperatures, β'_k, which are determined by the details of the process. Since no baths are present in this case, the irreversibility is linked to the non-adiabatic transitions between energy levels as a consequence of the process. Then, the backward part of the process starts from the resulting relaxed state. As no generalised heat can be transferred to external baths, the cycle ends with $\Delta\mathcal{F} = 0$ only if the process is slow enough to avoid any non-adiabatic transitions. Hence, the degree of irreversibility is measured by $\Delta\mathcal{F}$. If $\Delta\mathcal{F} > 0$, a part of the generalised mechanical work has been transformed into irreversible microscopic changes in the system.

QJE and our choice of inverse temperatures in the simulations

On the other hand, the QJE measures the fluctuations of $\mathcal{W}$ in the forward part of the process, and hence the (eventual) backward part is totally irrelevant. It is thus fundamental to note that the value $\mathcal{F}'$ used to obtain $\Delta\mathcal{F}$ is *not* the actual generalised free energy of the system after the forward part of the protocol, but the generalised equilibrium free energy evaluated with the final Hamiltonian, $\hat{H}'$, with temperatures $\vec{\beta}'$ (for a similar discussion concerning the standard QJE see, for example, the section 6.1 of [14]). In other words, the knowledge of the final equilibrium state after the forward part of the protocol, either arising from a connection to external baths, or from internal dynamics in isolation, is *not* required to apply QJE. This equality links a dynamical quantity which depends on the details of the protocol, the generalised mechanical work, with an equilibrium magnitude, the generalised free energy, that only depends on the initial equilibrium state, and the initial and final values of the external parameters of the Hamiltonian. The fact that both generalised QJE and TCR are formulated as functions of (possibly) different number of conserved charges, $\mathcal{N}$ and $\mathcal{N}'$, and (possibly) different temperatures, $\vec{\beta}$ and $\vec{\beta}'$, allows us to apply TCR to many different kinds of non-equilibrium processes, but it is irrelevant for the QJE. Hence, the most simple way to test the goodness of QJE in an experimental setup, and to study the existence of missing charges in the corresponding system, as we propose in the main part of the paper, is to take $\beta_k = \beta'_k \ \forall k$.

Supplementary Note 3
Review of the Dicke model and choice of simulation parameters

The Dicke model was formulated over 60 years ago to describe the interaction of an ensemble of N two-level atoms, with internal energy splitting $\hbar\omega_{\text{at}}$, with a monochromatic radiation field of frequency ω_{COM} [25, 26]. Its main feature is the transition from normal behaviour to super-radiance at a critical coupling g_{cr}, entailing a macroscopic population of the atomic excited state and the photon field, even at zero temperature [27–29]. Recent theoretical progress has highlighted its relevance to study excited-state quantum phase transitions [30]. For the purposes of this work, it is especially interesting the possibility to analyse a transition from integrability to chaos as a function of a single parameter, α [31–33]

In its most general formulation, the Hamiltonian of the model reads

$$\hat{H}(g,\alpha) = \hbar\omega_{\text{COM}}\hat{b}^\dagger\hat{b} + \hbar\omega_{\text{at}}\hat{J}_z + \frac{2\hbar g}{\sqrt{N}}\Big[(1-\alpha)\left(\hat{J}_+\hat{b} + \hat{J}_-\hat{b}^\dagger\right) + \alpha\left(\hat{J}_+\hat{b}^\dagger + \hat{J}_-\hat{b}\right)\Big]. \tag{6}$$

If $\alpha = 0$, the model is fully integrable; the quantity $\hat{M} = \hat{J} + \hat{J}_z + \hat{b}^\dagger\hat{b}$ is conserved. If $\alpha = 1$, the model is also fully integrable; in this case, the conserved quantity beyond the Hamiltonian is $\hat{M}' = \hat{J} + \hat{J}_z - \hat{b}^\dagger\hat{b}$. The integrability is broken for $0 < \alpha < 1$, though it has been recently shown that an approximated second integral of motion, specially in the low-energy region, exists even for $\alpha = 1/2$ [33]. Therefore, this model constitutes an ideal choice to test the new QFRs with initial GGE equilibrium states, by controlling the single parameter, α.

We can engineer a huge number of protocols, including:

(a) the exact conservation of the second integral of motion, for example a quench from $\hat{H} = \hat{H}(g_1, \alpha = 0)$ to $\hat{H}' = \hat{H}(g_2, \alpha = 0)$;

(b) the change from two different integrals of motions, like a quench from $\hat{H} = \hat{H}(g_1, \alpha = 0)$ to $\hat{H}' = \hat{H}(g_2, \alpha = 1)$; and

(c) the transition from integrable to non-integrable regimes, like quenches with $\hat{H} = \hat{H}(g_1, \alpha \in \{0, 1\})$ and $\hat{H}' = \hat{H}(g_2, 0 < \alpha < 1)$.

From this wide variety of possibilities, in the main part of the text we have studied a protocol of type (a). Specifically, we have chosen the following two-quench protocol: $(g_1 = 2\varepsilon_0, \alpha = 0) \to (g_2, \alpha = 1/2) \to (g_3 = \varepsilon_0, \alpha = 0)$, allowing the system to remain a time τ in the intermediate stage. With this choice, we have the same conserved quantity, $\hat{M}$, in both the initial and the final stages of the protocol. This choice has two main features. First, the initial stages for both the forward and the backward protocols share the same charge $\hat{M}$. And second, we can control whether $\hat{M}$ is (approximately) conserved during the protocol, just by changing the duration of the intermediate stage τ [34]. Thus, the results in the main part of the text highlight both the relevance of charges in work statistics, as well as the fact that the existence of charges at intermediate stages can be accounted for through the generalised QFRs – and not with the standard ones.

After the system is prepared in the initial state we proceed in the following way. First, we quench the system

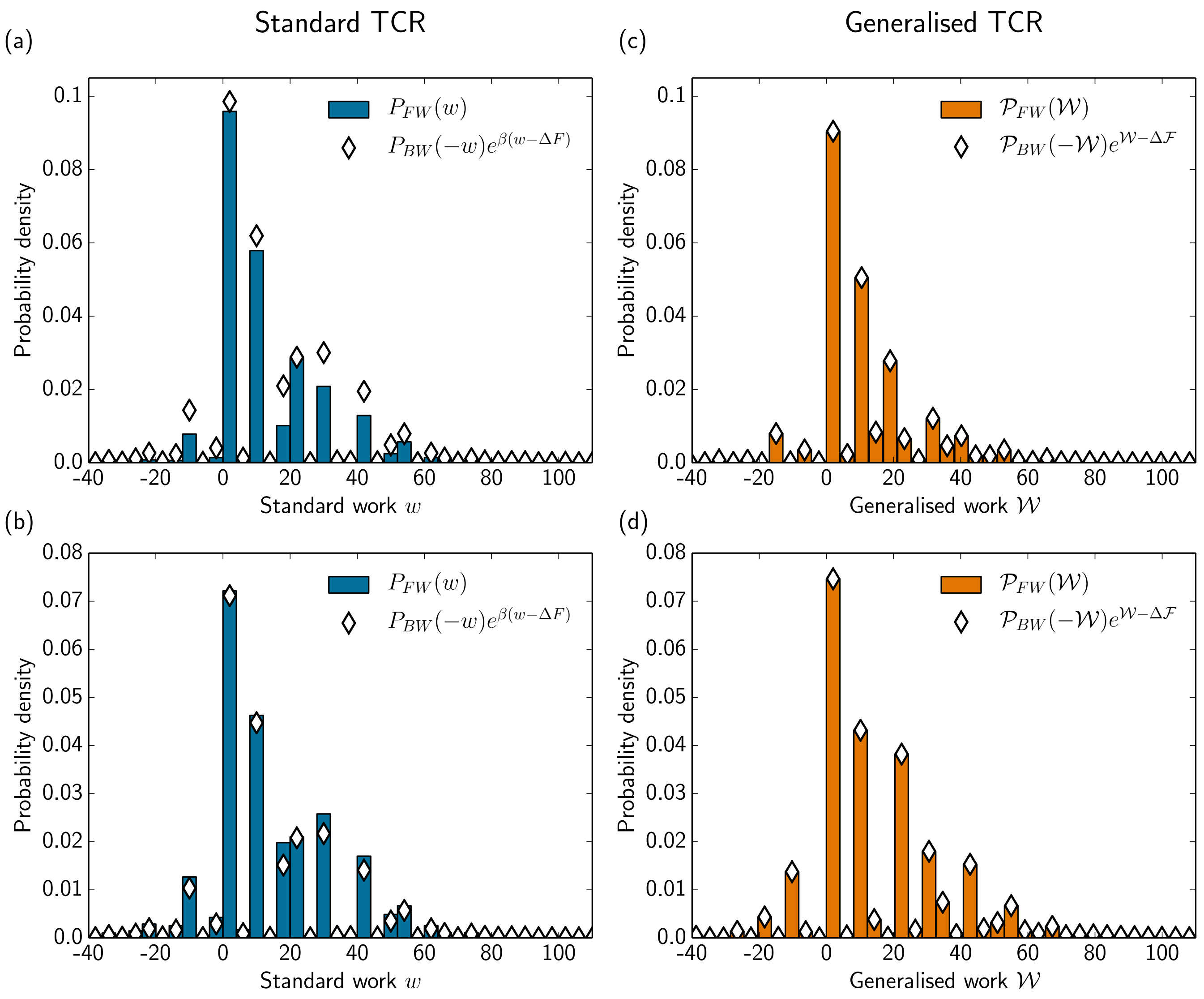

Supplementary Figure 1. Generalised Tasaki-Crooks relation with varying number of charges. Check of the standard and generalised Tasaki-Crooks relations using, respectively, standard work (panels a,b) and generalised work (panels c,d) for processes starting with $\alpha_{\text{FW}}(t=0)=0$ and ending at $\alpha_{\text{FW}}(\tau)=1/2$, where no additional exists. The protocol duration is $\tau = 1.024\ \mu$s, and initial equilibrium states are given by: (a,b) $(\beta\varepsilon_0, \beta_M\varepsilon_0) = (0.1, 0.3)$ and $\beta'\varepsilon_0 = 0.1$; (c,d) $(\beta\varepsilon_0, \beta_M\varepsilon_0) = (0.1, -0.1)$ and $\beta'\varepsilon_0 = 0.1$. Other parameters: $N = 7$ ions, $\omega_{\text{COM}} = 3\varepsilon_0$, $\omega_{\text{at}} = 10\varepsilon_0$, $g_{\text{ini}} = 2\varepsilon_0$, $g_{\text{int}} = 3\varepsilon_0$, and $g_{\text{fin}} = \varepsilon_0$, with $\varepsilon_0 = \hbar \times 2\pi$ MHz.

to the intermediate stage, $(g_2 = 3\varepsilon_0, \alpha = 1/2)$. We assume that the change in the externals parameters of the Hamiltonian is fast enough to disregard the explicit time dependence of the Hamiltonian; the great majority of the experiments dealing with non-equilibrium processes in small quantum systems are satisfactorily described in this way [35]. Then, we let the system relax in this intermediate stage by evolving with the new values of the parameters for times $0 < t < \tau$. As discussed above, the value of τ is critical to determine whether $\hat{M}$ is approximately conserved or not: the smaller the value of τ, the better the (approximate) conservation of $\hat{M}$ throughout the whole process. Finally, we perform a second quench to the final stage in the same way.

Supplementary Note 4
Fluctuations with a varying number of charges

In the main part of the article, we have discussed numerical results for a protocol involving quenches of the Dicke model, such that the initial Hamiltonian of both FW and BW processes is the same and features one additional charge, $\hat{M} = \hat{J} + \hat{J}_z + \hat{b}^\dagger \hat{b}$. To illustrate the power of the generalised QFRs to deal with situations where the total number of charges of the system changes as a result of the protocol, we show here results of a single-quench

protocol of type (c). Specifically, we consider the quench $(g = 2\varepsilon_0, \alpha = 0) \to (g = \varepsilon_0, \alpha = 1/2)$.

As $\hat{M}$ does not commute with $\hat{H}'$, it does evolve after the quench, and we focus on analysing the PDFs of work and generalised work for after a fixed evolution time, $\tau = 1.024\hbar/\varepsilon_0$. These results are shown in Supplementary Fig. 1(a,b), which correspond to an initial state of the FW process with $(\beta, \beta_M)\varepsilon_0 = (0.1, 0.3)$ or $(\beta, \beta_M)\varepsilon_0 = (0.1, -0.1)$, respectively (for simplicity, we take the initial state of the BW process to be given by $\beta' = \beta$ as well); these figures are in complete analogy to those in Fig. 2(c-f) of the main text. Again, we observe that the PDFs of generalised work for the FW and BW processes fulfil the generalised TCR, Eq. (4), while the PDFs of standard work noticeably disagree with the predictions from the standard TCR. Interestingly, for these simulations, we observe how the initial constraint on the allowed values of $\hat{M}$, is reflected even after the quench —i.e., when $\hat{M}$ no longer commutes with the Hamiltonian— in the PDFs of both generalised and standard work through vanishing probabilities for those values of, respectively, $\mathcal{W}$ and w that would relate to eigenstate transitions $|E_n, M_m\rangle \to |E'_p\rangle$ with initial states incompatible with the $\hat{M}$-constraint.

[1] C. Gogolin and J. Eisert, "Equilibration, thermalisation, and the emergence of statistical mechanics in closed quantum systems.", Rep. Prog. Phys. **79**, 056001 (2016) [DOI: 10.1088/0034-4885/79/5/056001].

[2] J. von Neumann, "Beweis des Ergodensatzes und des H-Theorems in der neuen Mechanik", Z. Phys. **57**, 30–70 (1929) [DOI: 10.1007/BF01339852]; English translation: "Proof of the ergodic theorem and the H-Theorem in quantum mechanics", Eur. Phys. J. H **35**, 201–237 (2010) [DOI: 10.1140/epjh/e2010-00008-5].

[3] S. Goldstein, J. L. Lebowitz, C. Mastrodonato, R. Tumulka, and N. Zanghi, "Normal Typicality and von Neumann's Quantum Ergodic Theorem", Proc. R. Soc. A Math. Phys. Eng. Sci. **466**, 3203–3224 (2009) [DOI: 10.1098/rspa.2009.0635].

[4] A. J. Short, "Equilibration of quantum systems and subsystems", New J. Phys. **13**, 053009 (2011) [DOI: 10.1088/1367-2630/13/5/053009].

[5] A. J. Short and T. C. Farrelly, "Quantum equilibration in finite time", New J. Phys. **14**, 013063 (2012) [DOI: 10.1088/1367-2630/14/1/013063].

[6] P. Reimann and M. Kastner, "Equilibration of isolated macroscopic quantum systems", New J. Phys. **14**, 043020 (2012) [DOI: 10.1088/1367-2630/14/4/043020].

[7] M. Rigol, V. Dunjko, V. Yurovsky, and M. Olshanii, "Relaxation in a completely integrable many-body quantum system: An ab initio study of the dynamics of the highly excited states of 1D lattice hard-core bosons", Phys. Rev. Lett. **98**, 050405 (2007) [DOI: 10.1103/PhysRevLett.98.050405].

[8] M. Rigol, V. Dunjko, and M. Olshanii, "Thermalization and its mechanism for generic isolated quantum systems.", Nature **452**, 854–858 (2008) [DOI: 10.1038/nature06838].

[9] R. V. Jensen and R. Shankar, "Statistical behavior in deterministic quantum systems with few degrees of freedom", Phys. Rev. Lett. **54**, 1879–1882 (1985) [DOI: 10.1103/PhysRevLett.54.1879].

[10] J. M. Deutsch, "Quantum statistical mechanics in a closed system", Phys. Rev. A **43**, 2046–2049 (1991) [DOI: 10.1103/PhysRevA.43.2046].

[11] M. Srednicki, "Chaos and quantum thermalization", Phys. Rev. E **50**, 888–901 (1994) [DOI: 10.1103/PhysRevE.50.888].

[12] H. Tasaki, "From quantum dynamics to the canonical distribution: General picture and a rigorous example", Phys. Rev. Lett. **80**, 1373–1376 (1998) [DOI: 10.1103/PhysRevLett.80.1373].

[13] M. Srednicki, "Thermal fluctuations in quantized chaotic systems", J. Phys. A. Math. Gen. **29**, L75–L79 (1996) [DOI: 10.1088/0305-4470/29/4/003].

[14] L. D'Alessio, Y. Kafri, A. Polkovnikov, and M. Rigol, "From quantum chaos and eigenstate thermalization to statistical mechanics and thermodynamics", Adv. Phys. **65**, 239–362 (2016) [DOI: 10.1080/00018732.2016.1198134].

[15] B. Pozsgay, M. Mestyán, M. A. Werner, M. Kormos, G. Zaránd, and G. Takács, "Correlations after quantum quenches in the XXZ spin chain: failure of the generalized Gibbs ensemble.", Phys. Rev. Lett. **113**, 117203 (2014) [DOI: 10.1103/PhysRevLett.113.117203].

[16] B. Wouters, J. De Nardis, M. Brockmann, D. Fioretto, M. Rigol, and J.-S. Caux, "Quenching the anisotropic Heisenberg chain: exact solution and generalized Gibbs ensemble predictions.", Phys. Rev. Lett. **113**, 117202 (2014) [DOI: 10.1103/PhysRevLett.113.117202].

[17] G. Goldstein and N. Andrei, "Failure of the local generalized Gibbs ensemble for integrable models with bound states", Phys. Rev. A **90**, 043625 (2014) [DOI: 10.1103/PhysRevA.90.043625].

[18] T. Prosen, "Open XXZ spin chain: Nonequilibrium steady state and a strict bound on ballistic transport", Phys. Rev. Lett. **106**, 217206 (2011) [DOI: 10.1103/PhysRevLett.106.217206].

[19] T. Prosen and E. Ilievski, "Families of quasilocal conservation laws and quantum spin transport", Phys. Rev. Lett. **111**, 057203 (2013) [DOI: 10.1103/PhysRevLett.111.057203].

[20] E. Ilievski, M. Medenjak, and T. Prosen, "Quasilocal Conserved Operators in the Isotropic Heisenberg Spin-1/2 Chain.", Phys. Rev. Lett. **115**, 120601 (2015) [DOI: 10.1103/PhysRevLett.115.120601].

[21] E. Ilievski and J. De Nardis, "Microscopic Origin of Ideal Conductivity in Integrable Quantum Models", Phys. Rev. Lett. **119**, 020602 (2017) [DOI: 10.1103/PhysRevLett.119.020602].

[22] Y. Guryanova, S. Popescu, A. J. Short, R. Silva, and P. Skrzypczyk, "Thermodynamics of quantum systems with multiple conserved quantities", Nat. Commun. **7**, 12049 (2016) [DOI: 10.1038/ncomms12049].

[23] N. Yunger Halpern, P. Faist, J. Oppenheim, and A. Winter, “Microcanonical and resource-theoretic derivations of the non-Abelian thermal state”, Nat. Commun. **7**, 12051 (2016) [DOI: 10.1038/ncomms12051].

[24] J. M. Hickey and S. Genway, “Fluctuation theorems and the generalized Gibbs ensemble in integrable systems.”, Phys. Rev. E **90**, 022107 (2014) [DOI: 10.1103/PhysRevE.90.022107].

[25] R. H. Dicke, “Coherence in Spontaneous Radiation Processes”, Phys. Rev. **93**, 99–110 (1954) [DOI: 10.1103/PhysRev.93.99].

[26] B. M. Garraway, “The Dicke model in quantum optics: Dicke model revisited.”, Philos. Trans. A. Math. Phys. Eng. Sci. **369**, 1137–1155 (2011) [DOI: 10.1098/rsta.2010.0333].

[27] K. Hepp and E. H. Lieb, “On the superradiant phase transition for molecules in a quantized radiation field: the Dicke maser model”, Ann. Phys. (N. Y). **76**, 360–404 (1973) [DOI: 10.1016/0003-4916(73)90039-0].

[28] Y. K. Wang and F. T. Hioe, “Phase transition in the Dicke model of superradiance”, Phys. Rev. A **7**, 831–836 (1973) [DOI: 10.1103/PhysRevA.7.831].

[29] H. J. Carmichael, C. W. Gardiner, and D. F. Walls, “Higher order corrections to the Dicke superradiant phase transition”, Phys. Lett. A **46**, 47–48 (1973) [DOI: 10.1016/0375-9601(73)90679-8].

[30] P. Pérez-Fernández, P. Cejnar, J. M. Arias, J. Dukelsky, J. E. García-Ramos, and A. Relaño, “Quantum quench influenced by an excited-state phase transition”, Phys. Rev. A **83**, 033802 (2011) [DOI: 10.1103/PhysRevA.83.033802].

[31] C. Emary and T. Brandes, “Quantum chaos triggered by precursors of a quantum phase transition: the Dicke model.”, Phys. Rev. Lett. **90**, 044101 (2003) [DOI: 10.1103/PhysRevLett.90.044101].

[32] C. Emary and T. Brandes, “Chaos and the quantum phase transition in the Dicke model”, Phys. Rev. E **67**, 066203 (2003) [DOI: 10.1103/PhysRevE.67.066203].

[33] A. Relaño, M. A. Bastarrachea-Magnani, and S. Lerma-Hernández, “Approximated integrability of the Dicke model”, EPL **116**, 50005 (2016) [DOI: 10.1209/0295-5075/116/50005].

[34] C. Neill, P. Roushan, M. Fang, Y. Chen, M. Kolodrubetz, Z. Chen, A. Megrant, R. Barends, B. Campbell, B. Chiaro, A. Dunsworth, E. Jeffrey, J. Kelly, J. Mutus, P. J. J. O’Malley, C. Quintana, D. Sank, A. Vainsencher, J. Wenner, T. C. White, A. Polkovnikov, and J. M. Martinis, “Ergodic dynamics and thermalization in an isolated quantum system”, Nat. Phys. **12**, 1037–1041 (2016) [DOI: 10.1038/NPHYS3830].

[35] J. Eisert, M. Friesdorf, and C. Gogolin, “Quantum many-body systems out of equilibrium”, Nat. Phys. **11**, 124–130 (2015) [DOI: 10.1038/nphys3215].
